# α-Synuclein fibril-induced paradoxical structural and functional defects in hippocampal neurons

**DOI:** 10.1186/s40478-018-0537-x

**Published:** 2018-05-01

**Authors:** Jessica M. Froula, Benjamin W. Henderson, Jose Carlos Gonzalez, Jada H. Vaden, John W. Mclean, Yumei Wu, Gokulakrishna Banumurthy, Linda Overstreet-Wadiche, Jeremy H. Herskowitz, Laura A. Volpicelli-Daley

**Affiliations:** 1 Department of Neurology, Center for Neurodegeneration and Experimental Therapeutics, University of Alabama at Birmingham, Birmingham, AL 35294 USA; 2 Department of Neurobiology, University of Alabama at Birmingham, Birmingham, AL 35294 USA; 3 Department of Neuroscience, Yale University School of Medicine, New Haven, CT 06510 USA; 4 Department of Cell Biology, Yale University School of Medicine, New Haven, CT 06510 USA; 5 Howard Hughes Medical Institute, Yale University School of Medicine, New Haven, CT 06510 USA; 6 Program in Cellular Neuroscience, Neurodegeneration and Repair, Yale University School of Medicine, New Haven, CT 06510 USA

**Keywords:** α-Synuclein, Lewy body, Lewy neurite, Dendritic spines, Fibril, Parkinson’s disease, Physiology, Calcium imaging

## Abstract

Neuronal inclusions composed of α-synuclein (α-syn) characterize Parkinson’s Disease (PD) and Dementia with Lewy bodies (DLB). Cognitive dysfunction defines DLB, and up to 80% of PD patients develop dementia. α-Syn inclusions are abundant in the hippocampus, yet functional consequences are unclear. To determine if pathologic α-syn causes neuronal defects, we induced endogenous α-syn to form inclusions resembling those found in diseased brains by treating hippocampal neurons with α-syn fibrils. At seven days after adding fibrils, α-syn inclusions are abundant in axons, but there is no cell death at this time point, allowing us to assess for potential alterations in neuronal function that are not caused by neuron death. We found that exposure of neurons to fibrils caused a significant reduction in mushroom spine densities, adding to the growing body of literature showing that altered spine morphology is a major pathologic phenotype in synucleinopathies. The reduction in spine densities occurred only in wild type neurons and not in neurons from α-syn knockout mice, suggesting that the changes in spine morphology result from fibril-induced corruption of endogenously expressed α-syn. Paradoxically, reduced postsynaptic spine density was accompanied by increased frequency of miniature excitatory postsynaptic currents (EPSCs) and presynaptic docked vesicles, suggesting enhanced presynaptic function. Action-potential dependent activity was unchanged, suggesting compensatory mechanisms responding to synaptic defects. Although activity at the level of the synapse was unchanged, neurons exposed to α-syn fibrils, showed reduced frequency and amplitudes of spontaneous Ca^2+^ transients. These findings open areas of research to determine the mechanisms that alter neuronal function in brain regions critical for cognition at time points before neuron death.

**Electronic supplementary material:**

The online version of this article (10.1186/s40478-018-0537-x) contains supplementary material, which is available to authorized users.

## Introduction

Neuronal inclusions composed of α-synuclein (α-syn) define Parkinson’s disease (PD) and Dementia with Lewy Bodies (DLB) [5]. Cognitive impairment characterizes DLB, and up to 80% of PD patients develop dementia [1, 35]. α-Syn inclusions are found in brainstem nuclei important for motor function, and are also abundant in the cortex and hippocampus where their formation predicts cognitive symptoms [3, 4, 9, 20, 21, 24, 56]. The role of α-syn in dopamine neurons of the substantia nigra pars compacta has been extensively studied because loss of these neurons causes the motor symptoms in PD. However, α-syn is expressed throughout the brain, primarily in excitatory, glutamatergic neurons in cortical and limbic brain regions [52]. Conversion of endogenous normal α-syn to pathologic aggregates may contribute to altered function in these neuronal subtypes which could impair cognition. Furthermore, in neurodegenerative diseases such as Alzheimer’s disease, synaptic dysfunction precedes neurodegeneration, and research is focused on identifying these early synaptic alterations [8, 45]. Identifying if and how synaptic dysfunction occurs in PD and DLB could point to novel therapeutic strategies to prevent or reverse neuronal defects and halt development of dementia.

α-Syn concentrates at the presynaptic terminal where it associates with synaptic vesicles and endosomes [7, 13, 32, 57], thus conversion of normal α-syn to pathologic aggregates could initially influence presynaptic function. Indeed, pathologic staging studies suggest that axonal Lewy neurites form before somal Lewy bodies and presynaptic aggregates of α-syn are highly abundant in DLB [9, 10, 26]. In fact, the contribution of abundant hippocampal Lewy neurites to dementia was appreciated well before the discovery that α-syn was the primary constituent of Lewy pathology [14]. Presynaptic aggregates of α-syn in the cortex of DLB patients correspond to reduced dendritic spines, suggesting they contribute to synapse loss and cognitive dysfunction [26]. Altering α-syn expression influences localization of synaptic vesicles at the active zone and neurotransmission [2, 18, 57, 63]. We previously showed that formation of pathologic inclusions sequesters α-syn away from the presynaptic terminal [62]. Thus, although multiple lines of evidence point to a role for α-syn at the presynaptic terminal and that presynaptic and axonal pathologic α-syn aggregates may contribute to neuronal dysfunction, it is unknown if formation of axonal α-syn inclusions impacts neuronal structure and function.

It has been difficult to study the impact of early formation of α-syn aggregates on neuron dysfunction because of the lack of suitable models. For example, in transgenic mice overexpressing mutant α-syn, aggregate formation is not apparent until the mice are several months old, and often coincides with neuronal death [19, 28, 33]. Overexpression of α-syn also does not faithfully produce aggregates that recapitulate those found in diseased brains. In contrast, the α-syn fibril model allows researchers to study inclusion formation from their initial formation. In this model, neurons are exposed to α-syn fibrils formed from recombinant α-syn that are internalized [25, 55, 62] and induce normal endogenous α-syn to form inclusions that biochemically and morphologically closely resemble those found in neurons of PD and DLB brains; they are insoluble in nonionic detergent, are filamentous by transmission electron microscopy and immuno-electron microscopy, are ubiquitinated, phosphorylated, bind p62 and exclude β-synuclein [29, 34, 38, 47, 54, 55, 62, 66]. Inclusion formation in primary neurons follows a lag phase of 2–4 days. By 4–7 days small, punctate aggregates form, and by 10–14 days after adding the fibrils, the aggregates grow and become more elongated, resembling Lewy Neurites, and can be found in approximately 30% of neuronal soma and dendrites where they appear skein-like, but over time form condensed accumulations that resemble Lewy Bodies [59, 62]. Formation of these aggregates causes defects in synchronous neuron firing, culminating in neuron death. Importantly, when fibrils are added to neurons from α-syn knockout mice, there is no phosphorylated α-syn, synchronous firing is similar to neurons not exposed to fibrils and there is no cell death [6, 29, 41, 62]. Thus the phenotypes produced result from corruption of endogenous α-syn to form insoluble, phosphorylated aggregates, and not from exogenous addition of fibrils to the neurons.

The goal of this study was to utilize the fibril model to analyze the impact of formation of α-syn inclusions on excitatory hippocampal neuronal function at a time point preceding neuron death. We found that formation of α-syn inclusions increased the frequency of presynaptic action potential-independent (miniature) mEPSCs with increased number of docked presynaptic vesicles, despite a major reduction in dendritic spine density. Spontaneous synaptic activity driven by action potentials remained normal, but there was a major impairment in spontaneous Ca^2+^ transients downstream of the synapse. These data suggest that corruption of endogenous α-syn into pathologic inclusions causes multiple defects in both pre- and post- synaptic function that begin before neurodegeneration.

## Materials and methods

### Animals

All experimental procedures involving animals were performed in accordance with Institutional Animal Care and Use Committee guidelines, were approved by the University of Alabama at Birmingham’s Institutional Animal Care and Use Committee, and were in accordance with the National Institutes of Health Guide for the Care and Use of Laboratory Animals (publication no. 80–23). Adult wild-type C57BL/6 J mice were obtained from Jackson laboratories and bred in house.

### Generation of mouse α-Syn fibrils and addition to neuronal cultures

Was performed as previously described [39, 61]. Endotoxin units were 0.02 ng/mL of endotoxin per well. Neurons were plated at 5.2 × 10^4^ cells per cm^2^. Neurons were exposed to 2 μg/mL final concentration of fibrils.

### Immunofluorescence

Immunofluorescence was performed as described previous [61]. The following primary antibodies were used: pS129-α-syn (Biolegend) and pS129-α-syn (EP1536Y, Abcam), NeuN (EMD Millipore), Tau (Dako), and Neurofilament heavy polypeptide (Abcam).

### Cell death assay

Neurons were rinsed once with phosphate buffered saline (PBS) and incubated with 0.8 μM calcein AM and 0.10 μM ethidium homodimer-I for 30 min. Cells were rinsed with PBS and examined with a Nikon Eclipse Ti-E inverted microscope. Using NIS Elements software, a tiled 5 × 5 image at 10X magnification was created for each treatment well and particles on each channel were counted using ImageJ.

### Spine density analysis

At 12 DIV, hippocampal neurons were transfected with Lifeact-GFP using Lipofectamine LTX (Invitrogen) according to manufacturer instructions. Two days after transfection, neurons were fixed with 4% paraformaldehyde and 4% sucrose in PBS, rinsed 3 times with PBS and mounted with Prolong Gold (Life Tecnhologies). A Nikon Eclipse Ti microscope was used to imageLifeact-GFP. Images were captured using a 60× oil-immersion objective. Z-series images were acquired at 0.15 μm increments through the entire visible dendrite. Automated image analysis was performed as described [51]. Briefly, Z-stack image series were deconvolved in Huygens using the blind algorithm that employs an iteratively refined theoretical point spread function. For preparation of figures, maximum intensity Z-projections were generated in Imaris. For quantitation, an ROI was selected containing a dendritic segment 40–60 μm in length that was distal to a dendritic branch point and void of crossing neurites or any additional dendritic branch points. Automatic thresholds were employed to assign dendrite end points and dendrite surface rendering. The maximum spine length and minimum spine end diameter were set at 15 μm and 0.215 μm, respectively. A trace was generated and a filter was applied to ensure that all dendritic protrusions ≤15 μm were assigned as spines. Dendritic protrusions were classified as described previously [22].

### Electrophysiology

Coverslips were mounted on a BX51WI Olympus microscope and superfused with recording solution containing (in mM): 140 NaCl, 2.8 KCl, 1 MgCl_2_, 1.5 CaCl_2_, 10 HEPES, 10 D-glucose/NaOH (pH 7.4). Cells were voltage clamped at − 70 mV with borosilicate electrodes (2–4 MΩ filled with an internal solution containing (in mM): 125 K-gluconate, 10 mM KCl, 10 HEPES, 2 Mg-ATP, 0.2 Na-GTP, 0.5 EGTA/KOH (pH 7.3). Electrodes were mounted on the headstage of a Multiclamp 700A amplifier (Molecular Devices, Union City, CA), allowing cancellation of capacitance transients and compensation of series resistance. Currents were filtered at 2 kHz and sampled at 10 kHz. Synaptic activity was recorded in the same neurons at room temperature (~ 24 °C) for 5 min in 3 conditions: control (no antagonists), 100 μM picrotoxin (PTX) to isolate glutamatergic synaptic currents, and 100 μM PTX with 500 nM TTX to isolate miniature EPSCs (mEPSCs). In control and PTX conditions, we detected and analyzed the rhythmic large bursts of synchronized compound events representing action-potential dependent activity using a threshold set at 100 pA; the high rate of activity precluded reliable detection of individual events. Note the spontaneous synaptic activity driven by action potentials and mEPSCs were recorded from the same neurons. Recordings with leak currents > 100 pA or series resistance > 20 MΩ were discarded. Data analysis was performed with the MiniAnalysis program (Synaptosoft, Leonia, NJ, USA) and Graphpad Prism (Graphpad, La Jolla, CA). Group comparisons were carried out using unpaired t-tests and cumulative frequency distributions were compared with Kolmogorov-Smirnov tests.

### Electron microscopy and synaptic vesicle quantification

Transmission electron microscopy was performed as described previously [64]. Primary neurons were grown on Thermanox plastic coverslips (Electron Microscopy Sciences, Hatfield, PA) and treated with either PBS or PFFs at 7 DIV. Neurons were fixed in 2% glutaraldehyde and 2 mM CaCl_2_ in 0.1 M NaCacodylate buffer, pH 7.4, and postfixed for 1 h in 1% OsO_4_ and 1% potassium ferrocyanide in 0.1 M NaCacodylate and 2 mM CaCl_2_ buffer. Following washes, samples were stained overnight at 4 °C with 2% uranylacetate then dehydrated with increasing percentages of EtOH. Samples were embedded with a 1:1 dilution of Epon and EtOH followed by pure Epon for baked for 48 h at 65C. Images were captured using a Tecnai Spirit T12 transmission electron microscope. 

### Live cell ca^+ 2^ imaging

Hippocampal neurons were treated with PBS, 2 μg/mL mouse α-syn monomer, or 2 μg/mL mouse α-syn fibrils at 7 DIV. Neurons were transduced with AAV9.Syn.GCamp6m.WPRE.SV40 at DIV 8 (University of Pennsylvania School of Medicine, Vector Core [12]. At DIV 14, spontaneous calcium activity was imaged at 37 °C in 136 mM NaCl, 2.5 mM KCl, 2 mM CaCl_2_, 1.3 mM MgCl_2_,10 mM glucose, and 10 mM 4-(2-hydroxyethyl)-1-piperazineethanesulfonic acid with a 10X objective, at 3 Hz acquisition using a Zeiss Cell Observer with a Colibri2 LED system. Movies were captured over 5 min. For each experiment, 4 fields were captured and 10 cell bodies were analyzed within each field. The average number of neurons in the fields of control neurons was 0.72 cm^2^ (+/− 0.009 cm^2^) and the average number of neurons in the fields of the fibril exposed neurons was 0.76 cm2 (+/− 0.03 cm^2^). Zeiss Zen software was used to calculate the fluorescence intensities every 300 msec over time. Custom-coded MATLAB scripts were used to analyze the amplitudes and frequencies of Ca^2+^ spikes.

## Results

### Formation of α-syn inclusions in axons

**Fig. 1:**
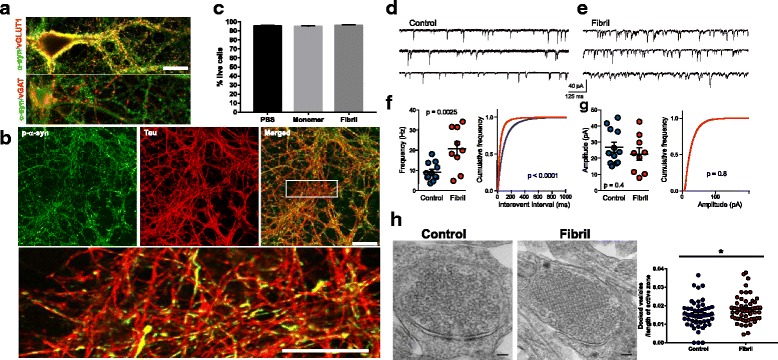
Seven days after exposure to α-syn fibrils, α-syn inclusions localize to axons, increase mEPSC frequency and increase the number of docked presynaptic vesicles. **a** Untreated primary hippocampal neurons were fixed at DIV 14. Immunofluorescence was performed with antibodies to total α-syn (green) and vGLuT1 (red) to label glutamatergic presynaptic terminals or vGAT1 (red) to label GABAergic terminals. Scale bar = 10 μm. **b**, **c** α-syn fibrils (2 μg/mL) were added to primary hippocampal neurons on DIV 7 and neurons were fixed 7 days later (DIV14). Immunofluorescence was performed using antibodies to p-α-syn (green) to label inclusions or tau (red) to label axons. Images were captured with a confocal microscope at an optical thickness of 0.5 μm. Scale bar = 50 μm, top panel, 25 μm, bottom panel. **c** Primary neurons were exposed to 2 μg/mL monomer, 2 μg/mL fibrils or PBS at DIV 7. Seven days later (DIV14), calcein AM was used to label live cells and ethidium homodimer-1 was used to label dead cells. Each well was scanned and tiled at 10X. Image J was used to quantify live and dead cells. A total of 32,527 PBS treated cells, 40,248 monomer treated cells, and 39,105 fibril treated cells were counted in two independent experiments. Data is expressed as the average live cells/total number of cells (sum of calcein positive and ethidium homodimer positive). *p* = 0.864 by ANOVA. **d**, **e** α-syn fibrils (2 μg/mL) were added to primary hippocampal neurons on DIV 7. Seven days later (DIV14) spontaneous synaptic activity was recorded in the presence of PTX, 100 μM, and TTX, 500 nM to isolate mEPSCs. Representative traces from control neurons and neurons treated with fibrils. **f** Average (+/− SEM) mEPSC frequencies and cumulative frequency plots of inter-event intervals for control neurons (blue, N-12) and neurons with inclusions (red, *N* = 9). *** represents *p* < .001 by independent t-test. **** *p* < 0.0001 by the Kolmogorov-Smirnov test. Experiments were performed in three independent coverslips. **g** Average (+/− SEM) mEPSC amplitude and cumulative frequency plot. **h** Representative images of presynaptic terminal from control hippocampal neurons and from hippocampal neurons 7 days after exposure to fibrils. Scale bar = 100 nm. The average length of the active zone was measured. The number of docked vesicles (≤ 50 nm from the plasma membrane) in excitatory presynaptic terminals was counted and data is expressed as average vesicles normalized to active zone length. Data was collected from two independent experiments. **** *p* < 0.05 by independent t-test

In primary hippocampal neurons, α-syn localized to presynaptic puncta that colocalized extensively with vGluT-1 in excitatory neurons with minimal overlap with the vesicular GABA transporter, vGAT (Fig. 1a) as demonstrated [53]. Inclusions localized to axons at initial time points after their formation which was induced by exposure of the neurons to α-syn fibrils generated from mouse α-syn (2 μg/mL) (Fig. 1b). p-α-Syn colocalized with tau, an axonal marker, at 7 days post fibril addition with minimal overlap with MAP2 in dendrites (Additional file 1: Figure S1A). Addition of monomeric α-syn did not produce p-syn positive inclusions (Additional file 1: Figure S1C). Another independent rabbit monoclonal antibody to p-α-syn [43], also showed inclusion localization to axons 7 days after initiation of inclusion formation (Additional file 1: Figure S1B,C). Initial formation of α-syn inclusions in axons and not the soma or dendrites is consistent with the predominant concentration of α-syn expression at the presynaptic terminal [13, 32, 57] and endosomes in axons [7].

At 7 days following exposure of primary neurons to fibrils, the percentage of live neurons was similar to control neurons treated with PBS or monomeric α-syn (Fig. 1c), as previously demonstrated using counts of NeuN-positive neurons in control and fibril treated cultures [62]. In contrast, 14 days after fibril exposure, there was a significant increase in the percentage of neurons with inclusions found in the soma, (Additional file 2: Figure S2A,B) with an approximately 30% reduction in the percentage of live neurons compared to PBS/control neurons (Additional file 2: Figure S2C). Thus, for the following experiments, we analyzed neurons at 7 days post-fibril exposure (DIV14) when inclusions are abundant, but there is no cell death.

We previously showed that the fibrils induce endogenously expressed α-syn to form inclusions that are not soluble in nonionic detergent such at TritonX-100 (Tx-100), similar to inclusions in PD brains [62]. To confirm these findings, neurons were sequentially extracted in Tx-100 followed by sodium dodecyl sulfate (SDS; insoluble fraction). In control neurons, α-syn was soluble in Tx-100 with no insoluble α-syn (Additional file 3: Figure S3A). In fibril treated neurons there was an appearance of α-syn in insoluble fraction. p-α-Syn was only present in the insoluble fraction in neurons exposed to fibrils. Immunofluorescence for p-α-syn was also apparent in neurons fixed with 4% paraformaldehyde with 1% Tx-100 to extract soluble proteins (Additional file 3: Figure S3B). Furthermore, α-syn inclusions morphologically appear similar to those found in PD brains [5, 10, 16, 49] such as dense spherical aggregates in the soma similar to Lewy bodies (Additional file 3: Figure S3C) and club-shaped aggregates similar to Lewy neurites. 

### Changes in presynaptic function caused by α-syn inclusions

To determine if pre- or postsynaptic function is altered in neurons with α-syn inclusions, we performed electrophysiology experiments to record mEPSCs in primary hippocampal neurons. To isolate mEPSCs of glutamatergic synapses, the inhibitors picrotoxin (PTX) and tetrodotoxin (TTX) were used. mEPSCs reflect synaptic activity in the absence of action potentials, resulting from the spontaneous release of single synaptic vesicles. Changes in the amplitude of mEPSCs reflect altered number of postsynaptic glutamate receptors, whereas changes in the frequency of mEPSCs reflect alterations in the number of synapses or the release probability of presynaptic vesicles. On DIV 7, primary hippocampal neurons were exposed to fibrils (or PBS as a control) and recordings were performed 7 days later (DIV14, a time point when there is no cell death) (Fig. 1c) in the presence of both picrotoxin (PTX) and tetrodotoxin (TTX) to isolate glutamatergic mEPSCs. There was a robust, significant increase in the frequency of mEPSCs (Fig. 1d, e, f) in fibril treated neurons quantified both as an increase in the average mEPSC frequency per cell and a reduction in the interevent interval, when events from all cells were combined. However, there was no significant change in the mEPSC amplitude (Fig. 1g). Thus, these data suggest that initial formation of α-synuclein inclusions increases presynaptic activity.

### Ultrastructure of the presynaptic terminal in neurons with α-syn inclusions

To further probe for presynaptic alterations, we performed transmission electron microscopy to determine if the increased mEPSCs resulted from increased abundance of presynaptic vesicles. Seven days after fibril or control treatment, primary hippocampal neurons were processed for transmission electron microscopy. Only presynaptic terminals at excitatory, asymmetric synapses were quantified [40] (Fig. 1h). There were no significant differences in the length of the active zone or total number of synaptic vesicles between control neurons and neurons with inclusions (Additional file 4: Figure S4). However, there was a slight but significant increase in the number of docked synaptic vesicles per active zone in the fibril exposed neurons, which could at least in part cause the increased frequency of mEPSCs (Fig. 1h). In addition, the synaptic vesicles often appeared aligned and clustered, similar to previous findings in syn knockout mice [57]. It has previously been shown that synuclein knockout neurons have increased docking of synaptic vesicles which correlates with increased phosphorylation of synapsin I, which plays a role in regulating the synaptic vesicle reserve pool. We also previously showed that 14 days after exposure of neurons to fibrils, the SNARE proteins syntaxin 1 and VAMP2 are reduced. We thus tested whether levels of synapsin I or SNARE proteins were altered 7 days after exposure of neurons to fibrils. Quantitation of immunoblots showed no significant differences in levels of SNARE proteins, synapsin I or phosphorylation of synapsin I at serine 62 and serine 67 in neurons 7 days after induction of inclusion formation compared to controls (Additional file 5: Figure S5). Therefore, the increased docking and changes in synaptic vesicle organization cannot be caused by significant changes in levels or phosphorylation of these synaptic proteins. Overall, these data suggest that increased mEPSCs result from docking of presynaptic vesicles.

### Changes in dendritic spine density and morphology in neurons caused by corruption of endogenous α-syn

**Fig. 2:**
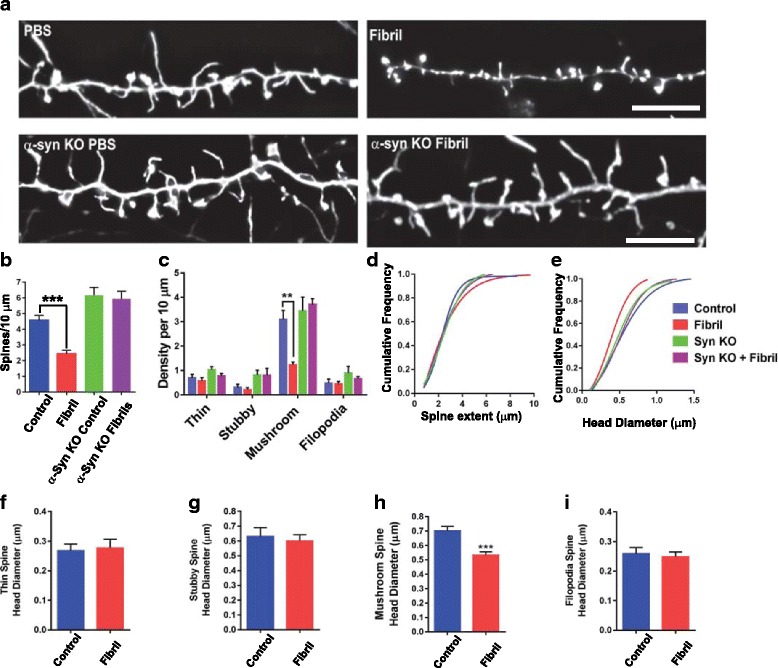
Formation of α-syn inclusions in primary hippocampal neurons reduces density and head diameter of mushroom-shaped dendritic spines. Primary hippocampal neurons from wild type mice or α-syn knockout mice were exposed to fibrils or PBS at DIV 7. On DIV 12, neurons were transfected with LifeAct-GFP. Two days later (DIV 14, 7 days after adding fibrils), neurons were fixed. Widefield microscopy was used to capture Z-stacks of spines, followed by image deconvolution. **a** Representative images of dendritic spines in control neurons (top left), neurons 7 days after fibril induction of inclusion formation (top right). The bottom 2 panels are representative images from neurons lacking endogenous α-syn without (bottom left) or with exposure to fibrils (bottom right). Scale bars equal 5 μm. **b** The number of spines per 10 μm dendritic length was quantified in wild type, control neurons (blue bar; *N* = 7), wild type neurons after exposure to fibrils for 7 days (red bar; *N* = 7), α-syn knockout, control neurons (green bar; *N* = 6) and α-syn knockout neurons exposed to fibrils for 7 days (purple bar; *N* = 6). Data represents the means +/− SEM from two independent coverslips. F = 23.5, *p* < .0001 by ANOVA with Dunnett’s post-hoc test. **c** The number of thin, stubby, mushroom or filopodia shaped spines were quantified per 10 μm dendritic length. Two way ANOVA F = 5.85 (interaction), F = 17.41 (treatment) ** represents *p* < .0001. **d** Cumulative frequency plot of spine extent (length). *P* = 0.95, Kruskal-Wallis test. **e** Cumulative frequency plot of spine head diameter. *P* = 0.001 Kruskal-Wallis test. **f** Spine head diameter of thin, stubby, mushroom or filopodia shaped spines was quantified for wild type control neurons and neurons with α-syn inclusions. *** represents *p* < .0001 by independent t-test

Excitatory presynaptic terminals synapse on dendritic spines. Spine density and morphology correlate with synaptic strength and activity [23]. To analyze how spine morphology is altered by formation of α-syn inclusions, on DIV7, primary hippocampal neurons were exposed to fibrils (or PBS as a control), and 5 days later transfected with Lifeact-GFP which allows visualization of filamentous actin without interfering with actin dynamics [42]. Two days later, (7 days following initiation of inclusion formation; DIV 14), neurons were fixed and spine morphometry analyses was performed. There was a significant, approximately 50%, reduction in spine density in neurons 7 days following induction of inclusion formation with α-syn fibrils (Fig. 2a, b). Importantly, when fibrils were added to α-syn knockout (KO) neurons that do not express endogenous α-syn, the density of dendritic spines was equivalent to control neurons (Fig. 2a, b). This demonstrates that fibrils themselves did not perturb dendritic morphology, but rather suggests that corruption of endogenous α-syn caused reduced spine density.

Spines can be classified based on structure into thin, stubby, or mushroom [23]. Analysis of the density of the structures showed that stable mushroom spines are significantly and selectively reduced in neurons with α-syn inclusions (Fig. 2c). Again, neurons from α-syn KO mice exposed to fibrils showed no changes in the density of any spine sub-classes. The overall length or extent of spines was not altered in neurons with α-syn inclusions (Fig. 2d). There was a significant reduction of mushroom spine head diameter in neurons with α-syn inclusions (Fig. 2e, h) with no changes in head diameter in thin, stubby spines or filopodia (Fig. 2f, g, i). Overall, these data demonstrate that corruption of endogenous α-syn at a time point preceding neuron death results in a major reduction in mushroom spine density.

Cell surface biotinylation and immunoblots to investigate levels of surface and total levels of the NR2A and NR2B of NMDA receptor subunits and the GluR1 and GluR2 AMPA receptor subunits revealed no significant changes between control neurons and neurons with α-syn inclusions (Additional file 6: Figure S6). The results of the cell surface pull-down assays thus demonstrate that although there is a major reduction in spine density, there is no change in the overall cell surface expression of NMDA and AMPA receptor subunits. Overall, these data suggest that major reductions in spine density are an early phenotype caused by formation of α-syn inclusions.

### Spontaneous synaptic activity driven by action potential dependent and independent glutamate release in neurons 7 days following exposure to fibrils

**Fig. 3:**
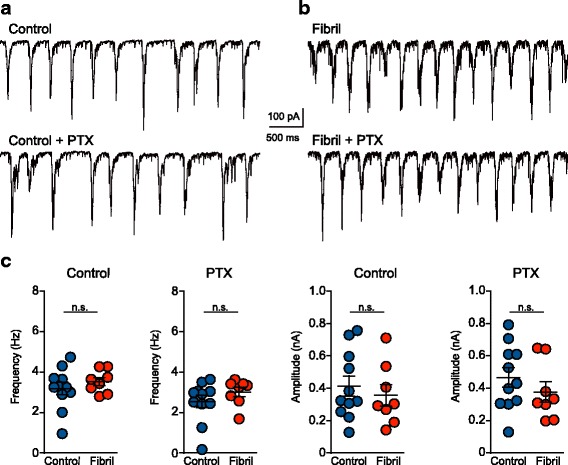
Spontaneous synaptic activity driven by action potentials is normal in neurons with α-syn inclusions. EPSCs were analyzed over 10 min in neurons 7 days following exposure to fibrils (DIV14) or control neurons in the absence or presence of PTX, 100 μM, to isolate excitatory currents. sEPSCs were recorded from the same neurons as the mEPSC recordings in Fig. 1. Burst events were detected with a threshold of 100 pA. There were no significant differences in the frequency or amplitude of burst events. Unpaired t-tests, control cells (*N* = 11) and fibril-treated neurons (*N* = 8)

Given the major reduction in dendritic spines and increased mEPSC frequency, neurons 7 days after exposure to fibrils likely show overall alterations in action potential-driven events resulting from changes in presynaptic activity or overall network activity. To test this, we recorded action-potential dependent synaptic activity from primary hippocampal neurons 7 days after exposure to fibrils. Recordings were performed both in the presence or absence of picrotoxin to isolate spontaneous EPSCs (the same neurons were also recorded in picrotoxin and TTX for experiments in Fig. 1). We focused on large compound spontaneous events that represent bursts of synaptic activity resulting from semi-synchronized activation of multiple neurons, setting a threshold to detect events larger than three times mEPSC average amplitude (~ 100 pA). This patterned activity was absent in TTX, confirming that it requires action potentials. Surprisingly, there were no major differences in the frequency or amplitude of spontaneous EPSCs between control neurons and neurons with inclusions (Fig. 3). These data suggest that even with increased mEPSC frequency and reduced spine density, compensatory mechanisms can maintain normal levels of spontaneous synaptic activity driven by action potentials.

### Effect of α-syn inclusions on spontaneous calcium transients in hippocampal neurons

**Fig. 4:**
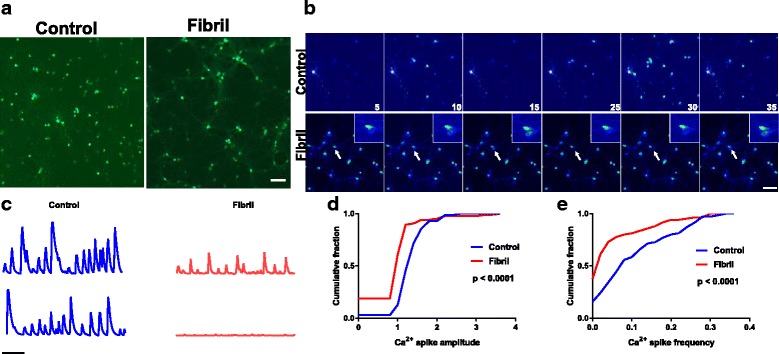
Formation of α-syn inclusions impair spontaneous Ca^2+^ transients. Primary hippocampal neurons from wild type mice were exposed to fibrils (or PBS) at DIV 7. On DIV 8, primary neurons were transduced with AAV9-Synapsin1-GCamp6 to visualize Ca^2+^ transients selectively in neurons. On DIV 14, (7 days post induction of inclusions with fibrils), neurons were imaged live. Images were captured every 300 msec for 5 min at 37 °C. **a** Images of neurons expressing AAV9-Synapsin1-GCamp6. Scale bar = 100 μm. **b** Representative images of Ca^2+^ transients (pseudocolored so red represents high levels of GCamp6 fluorescence and blue represents low levels of GCamp6 fluorescence.) **c** Representative traces of Ca^2+^ transients over time in control neurons and neurons with inclusions. **d** Custom Matlab scripts quantified the frequency of Ca^2+^ spikes and amplitude of the Ca^2+^ spikes. Cumulative frequency plots show frequency and amplitude of spikes in control neurons (blue) and neurons with inclusions (red). Data was collected from three independent experiments. **** *p* < 0.0001 by the Kolmogorov-Smirnov test

Another role of dendritic spines, in addition to receiving synaptic inputs, is compartmentalization of calcium [31, 65]. To determine if α-syn inclusions cause defects in Ca^2+^ signaling, primary hippocampal neurons were exposed to fibrils for 7 days (total DIV14). At DIV8, neurons were transduced with AAV9-Synapsin-GCaMP6m which allows Ca^2+^ imaging selectively in neurons [12]. Spontaneous Ca^2+^ transients were imaged live for 5 min. Figure 4a and b show representative images of AAV9-Synapsin-GCaMP6m in control neurons and in neurons 7 days following induction of inclusion formation. Control neurons show robust Ca^2+^ transients. In fibril-exposed neurons, some neurons show Ca^2+^ transients, but others show no Ca^2+^ activity. Quantitation of Ca^2+^ transients in the neuronal soma over time results in an average frequency of 0.1 spikes per second (±0.01) in control neurons and of 0.05 spikes per second (±0.008) in neurons 7 days after fibril exposure. The maximum average fluorescence intensity in control neurons was 1.4 (±0.05) and was 1.0 (±0.07) for fibril exposed neurons, respectively. Cumulative frequency plots show that in neurons 7 days following induction of inclusion formation the frequency of Ca^2+^ transients and the amplitude of Ca^2+^ transients were significantly reduced (Fig. 4c, d). Therefore, although the is no cell death at 7 days after exposure of neurons to fibrils, there is a robust decrease in Ca^2+^ demonstrating major defects in neuron function preceding neuron loss.

## Discussion

We showed that pathologic α-syn inclusions cause alterations in synaptic function of excitatory hippocampal neurons at a time point before cell death. When α-syn inclusions primarily localize to axons (DIV 7), neurons show a paradoxical increase in presynaptic activity with a reduction in the density of stable, mushroom spines. The overall lack of a change in spontaneous synaptic activity driven by action potentials suggests that the neurons compensate for these defects, at least at the level of the synapse. However, the neurons show reduced Ca^2+^ handling downstream of synaptic activity. This study provides additional endpoints for researchers to assess the potential of a therapeutic target in reversing neuron these alterations: spine morphology, physiology, Ca^2+^ imaging, in addition to impaired axonal transport of early endosomes as we demonstrated previously [60]. Importantly, the synaptic alterations in excitatory hippocampal neurons may be relevant for dementia and cognitive dysfunction in PD and DLB. Finally, future studies into the mechanisms by which α-syn inclusions cause these synaptic changes may reveal novel strategies to prevent or reverse these defects before intractable neuron death.

α-Syn inclusion initiation in axons is consistent with the presynaptic enrichment of endogenous α-syn [7, 32, 57] and pathological PD staging showing that Lewy neurite formation precedes Lewy body formation [9]. In addition, Lewy neurites and presynaptic α-syn aggregates are far more abundant than Lewy bodies [10, 16], highlighting the importance of understanding how pathologic accumulation of α-syn in the presynaptic terminal and axon influences neuronal function. At the presynaptic terminal, recruitment of normal α-syn into pathologic aggregates increases the frequency of mEPSCs. Typically increased mEPSC frequency results from either more synapses, increased release probability of vesicles at individual synapses, or changes in the synapse size. The quantitation of electron microscopy images revealed no changes in the size of presynaptic terminals. Changes in the pH or Ca^2+^ levels could also increase excitotoxicity in the culture resulting in elevated mEPSC frequency, although we did not find significant changes in the sEPSC activity arguing against this possibility. Remarkably, the robust increase in mEPSC frequency occurs despite the major reduction in spine density. Thus, increased mEPSC frequency likely results from increased availability of synaptic vesicles at remaining synapses to the docking site for release, and/or increased fusion probability of synaptic vesicles with the plasma membrane. Absence of α-syn in neurons increases presynaptic release [2, 46] and tethering of vesicles to the active zone [57]. Thus, initial recruitment of α-syn into inclusions may recapitulate a loss of α-syn function phenotype. Although we have not determined the mechanism by which induction of pathologic α-syn aggregation may increase presynaptic release, α-syn interacts with synapsin I and actin [27, 44, 48] which is important for trapping synaptic vesicles into the reserve pool. α-Syn normally acts as a brake on fusion of SNARE complexes and merging of synaptic vesicle membranes with the plasma membrane [63]. Recruitment of α-syn away from the presynaptic terminal into pathologic aggregates may release this brake. In addition, α-syn acts as a chaperone for SNARE complex assembly [11]. The increased release of synaptic vesicles may also result from pore formation caused by the α-syn fibrils [58]. Future studies of how sequestration of endogenous α-syn away from the presynaptic terminal impacts SNARE complex assembly and synaptic vesicle release are therefore critical.

Our findings show that corruption of endogenous α-syn causes a reduction in the density and head diameter of dendritic mushroom spines at a time point preceding cell death. Thus, Lewy body dementias may be similar to Alzheimer’s disease in that synaptic dysfunction precedes synapse loss which in turn precedes neurodegeneration. These findings support other studies showing that α-synucleinopathy reduces spine density in the cortex and that this may be a pathophysiological phenotype that contributes to dementia [6, 26]. It is possible that the reduced spines are homeostatic response to the increase presynaptic activity. It is also possible that abnormal aggregates of α-syn that are below our current methods of detection localize to dendrites and spines where they alter spine dynamics. Recent studies show that α-syn regulates actin dynamics through an interaction with spectrin. Dendritic spine morphology is regulated by actin dynamics and abnormal α-syn aggregates may perturb the actin cytoskeleton resulting in loss of spines [36].

The lack of a change in spontaneous synaptic events could be a combined result of increased presynaptic activity with reduced dendritic spines. Although there were no changes in spontaneous activity at the synaptic level, the frequency and amplitude of spontaneous Ca^2+^ spikes were reduced contributing to other research showing that α-syn perturbs Ca^2+^ homeostasis [37]. It is important to note that the somatic reductions in Ca^2+^ transients likely are not related to the increased frequency of mEPSCs. Recently it was shown that PD associated mutations in phospholipase A2 group 6 (PLA2g6) impair store operated Ca^2+^ entry and reduce stores of ER Ca^2+^ [67]. Abnormal α-syn also causes ER stress which could impact function of IP3 receptors, ryanodine receptors or sarco/endoplsmic reticular Ca^2+^-ATPases involved in release of Ca^2+^ from the ER.

It is important to note that we and others [52, 53] show that synuclein localizes primarily to glutamatergic terminals in the hippocampus, thus in this study, we focused on glutamatergic neurons. We also did not see differences between spontaneous activity when GABAA receptors were intact verses blocked, suggesting there were not major changes (that is, sEPSCs in control and in picrotoxin were similar between treated and un-treated neurons). However, future interesting studies could examine the impact of inclusions on GABAergic neuron structure and physiology, particularly in brain regions in which α-syn is expressed in GABAergic neurons such as the olfactory bulb, globus pallidus and substantia nigra pars reticulata [52].

In this paper, we used the α-syn fibrils as a model to examine the functional effects of misfolded α-syn within a neuron. However, we did not address the neuron to neuron spread of extracellular α-syn aggregates which contributes to disease progression [30, 50]. Acute treatment of hippocampal slices with soluble α-syn oligomers, but not fibrils or monomer, disrupts long term potentiation and causes synaptic dysfunction, which is mediated by oligomer association the cellular prion protein [15, 17]. Thus, the release and cell to cell propagation of α-syn likely further contributes to neuron dysfunction. These studies also suggest that the neuron dysfunction caused by oligomeric α-syn could play a role in the uptake and further trans-synaptic spread of α-syn.

Treating the nonmotor symptoms of PD and DLB is an unmet need. These findings demonstrate that α-syn inclusions cause neuronal dysfunction in hippocampal neurons which is relevant for cognitive decline in these disorders. These synaptic defects may also promote the “spread” of fibrillar α-syn seeds throughout vulnerable networks. Finally, our data highlight the need to develop tools to detect and remove abnormal α-syn at early stages of disease development. It is possible that by the time insoluble, phosphorylated α-syn inclusions form, the pathway toward neurodegeneration cannot be reversed.

## Conclusions

This paper demonstrates that formation of α-syn inclusions induces structural and functional alterations in excitatory neurons before neurodegeneration occurs, suggesting that neurons could be rescued before they die. These changes include increased presynaptic activity, loss of stable dendritic spines, and reduced Ca^2+^ transients. Formation of limbic and cortical α-syn inclusions correlates with dementia in PD and DLB. The defects found in this study suggest potential mechanisms in which intrinsic neuronal defects could contribute to cognitive dysfunction.

## Additional files

Additional file 1: **Figure S1. A.** Primary hippocampal neurons from DIV were fixed and immunofluorescence was performed using antibodies to PSD95 as a postsynaptic marker (red) and VAMP2 as a presynaptic marker (green). Colocalization of the presynaptic terminal and post synaptic density can be seen as yellow in the merged images. Arrows point to examples of mature synapses. Scale bar = 20 μm. **B.** Control α-Syn fibrils (2 μg/mL) were added to primary hippocampal neurons on DIV 7 and neurons were fixed 7 days later (DIV14). Immunofluorescence was performed using antibodies to p-α-syn (mouse antibody, green) to label inclusions or MAP2 (red) to label dendrites. Images were captured with a confocal microscope at an optical thickness of 0.5 μm. Scale bar = 10 μm. **C.** Immunofluorescence was performed using antibodies to p-α-syn (rabbit antibody, green) to label inclusions or neurofilament (red) to label axons. **D.** α-Syn monomer (2 μg/mL) was added to primary hippocampal neurons on DIV 7 and neurons were fixed 7 days later. Immunofluorescence was performed using antibodies to p-α-syn (mouse or rabbit antibody, green) to label inclusions or tau or neurofilament (red) to label axons. (PDF 922 kb)

Additional file 2: **Figure S2.** α-Syn fibrils (2 μg/mL) were added to primary hippocampal neurons on DIV 7 and neurons were fixed 14 days later. **A.** Immunofluorescence was performed using antibodies to p-α-syn (mouse antibody, green) to label inclusions, tau (red) to label axons, or Hoechst (blue) for nuclei. Images were captured with a confocal microscope at an optical thickness of 0.5 μm. Scale bar = 10 μm. Arrows point to examples of p-α-syn inclusions in the soma. B. The percentage of cells with inclusions near the nucleus was quantified (*N* = 9). Independent t-test *t* = 7.1, *p* < 0.0001. **C**. Primary neurons were exposed to 2 μg/mL monomer, 2 μg/mL fibrils or PBS at DIV 7. Fourteen days later, calcein AM was used to label live cells and ethidium homodimer-1 was used to label dead cells. Each well was scanned and tiled at 10X. Image J was used to quantify live and dead cells. A total of 40,663 PBS treated cells, 42,348 monomer treated cells, and 45,271 fibril treated cells were counted in two independent experiments. Data is expressed as the average live cells/total number of cells (sum of calcein positive and ethidium homodimer positive). *p* = 0.864 by ANOVA. (PDF 534 kb)

Additional file 3: **Figure S3. A.** Neurons were exposed to fibrils or PBS as a control and were sequentially extracted in 1% Tx-100 followed by 2%SDS. Lysates were subjected to SDS-PAGE on a 4–20% gel and immunoblots were performed with antibodies to p-α-syn, total α-syn or Tuj1 as a loading control. **B.** Neurons were exposed to fibrils and either fixed with 4% paraformaldehyde (left panel) or 4% paraformaldehyde with 1% Tx-100 (right panel). Immunofluorescence was performed with an antibody to p-α-syn. **C.** Confocal image of a dense spherical inclusion labeled using an antibody to p-α-syn. Hoechst shows the presence of nuclei, although the nucleus juxtaposed to the inclusion appears fainter compared to the healthier nuclei nearby. **D.** Examples of aggregates that appear morphologically similar to Lewy neurites. Scale bar = 100 μm. (PDF 919 kb)

Additional file 4: **Figure S4.** The length of the active zone and number of synaptic vesicles normalized to active zone length were quantified using transmission electron microscopy images. Only asymmetric synapses were quantified. (PDF 188 kb)

Additional file 5: **Figure S5.** Primary neurons were either untreated, treated with monomeric α-syn or α-syn fibrils. Seven days later cells were lysed and immunoblots were performed for total levels of syntaxin 1, VAMP2, SNAP25, Synapsin 1, or phospho-Synapsin 1 (site 4/5). The quantitation on the right shows levels of each protein normalized to loading control (vinculin for synapsin 1 and Synapsin 1 for other proteins). The control and fibril exposed neurons represent 6 independent experiments and the monomer exposed neurons represent 3 experiments. There were no significant differences by independent t-test. (PDF 134 kb)

Additional file 6: **Figure S6.** Primary hippocampal control neurons and neurons with inclusions (7 days post-fibril exposure) were incubated with biotin, lysed, and cell surface proteins were pulled down with neutravidin beads. The left immunoblots show cell surface NR2A, NR2B NMDA receptor subunits and GluR1 and GluR2 receptor subunits. The immunoblots on the right show total levels of each protein. Vinculin was included to demonstrate equal loading. Quantitation on the right from 6 independent experiments show the mean level of surface receptor subunits normalized to total levels of each protein. There were no significant differences by independent t-test. (PDF 129 kb)

## Abbreviations

DLB: Dementia with Lewy bodies
mEPSCs: Miniature excitatory postsynaptic potentials
PBS: Phosphate buffered saline
PD: Parkinson’s disease
PTX: Picrotoxin
TTX: Tetrodotoxin
α-syn: α-synuclein
