# Supplementary figures and images for: α-Synuclein fibril-induced paradoxical structural and functional defects in hippocampal neurons
Source: Acta Neuropathol Commun. 2018 May 1;6:35. doi: 10.1186/s40478-018-0537-x (PMC5928584; doi:10.1186/s40478-018-0537-x)

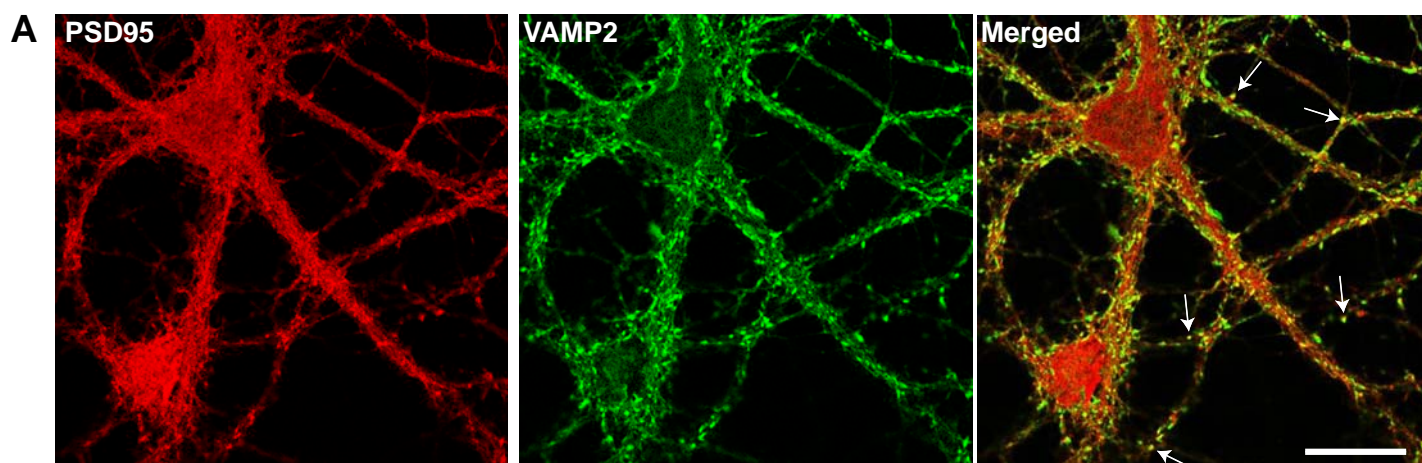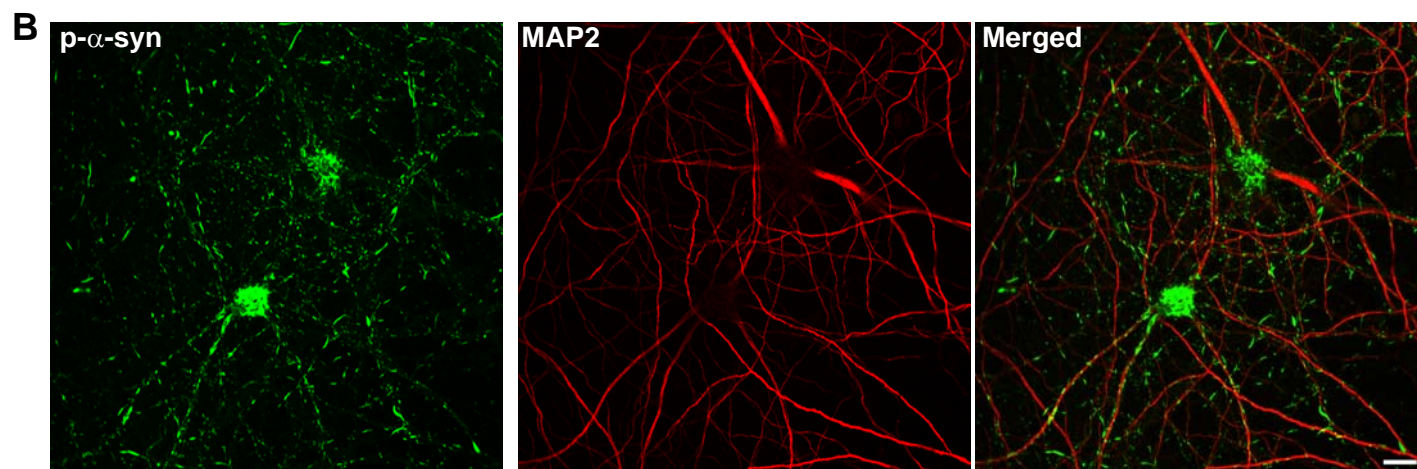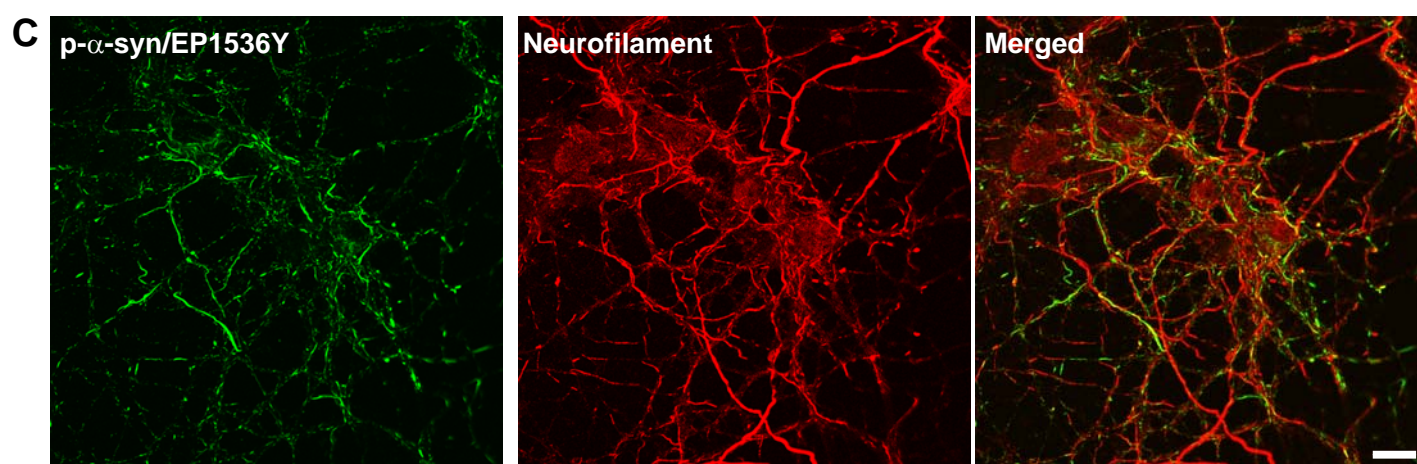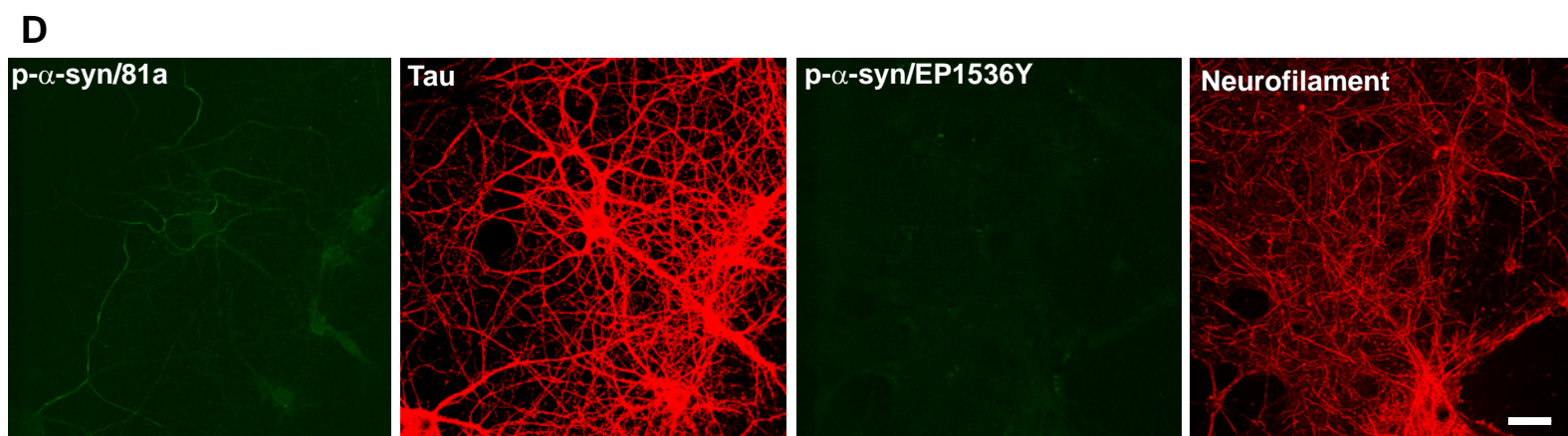

Supplement: Supplementary file 1 — Figure S1. A. Primary hippocampal neurons from DIV were fixed and immunofluorescence was performed using antibodies to PSD95 as a postsynaptic marker (red) and VAMP2 as a presynaptic marker (green). Colocalization of the presynaptic terminal and post synaptic density can be seen as yellow in the merged images. Arrows point to examples of mature synapses. Scale bar = 20 μm. B. Control α-Syn fibrils (2 μg/mL) were added to primary hippocampal neurons on DIV 7 and neurons were fixed 7 days later (DIV14). Immunofluorescence was performed using antibodies to p-α-syn (mouse antibody, green) to label inclusions or MAP2 (red) to label dendrites. Images were captured with a confocal microscope at an optical thickness of 0.5 μm. Scale bar = 10 μm. C. Immunofluorescence was performed using antibodies to p-α-syn (rabbit antibody, green) to label inclusions or neurofilament (red) to label axons. D. α-Syn monomer (2 μg/mL) was added to primary hippocampal neurons on DIV 7 and neurons were fixed 7 days later. Immunofluorescence was performed using antibodies to p-α-syn (mouse or rabbit antibody, green) to label inclusions or tau or neurofilament (red) to label axons. (PDF 922 kb) [file 40478_2018_537_MOESM1_ESM.pdf]

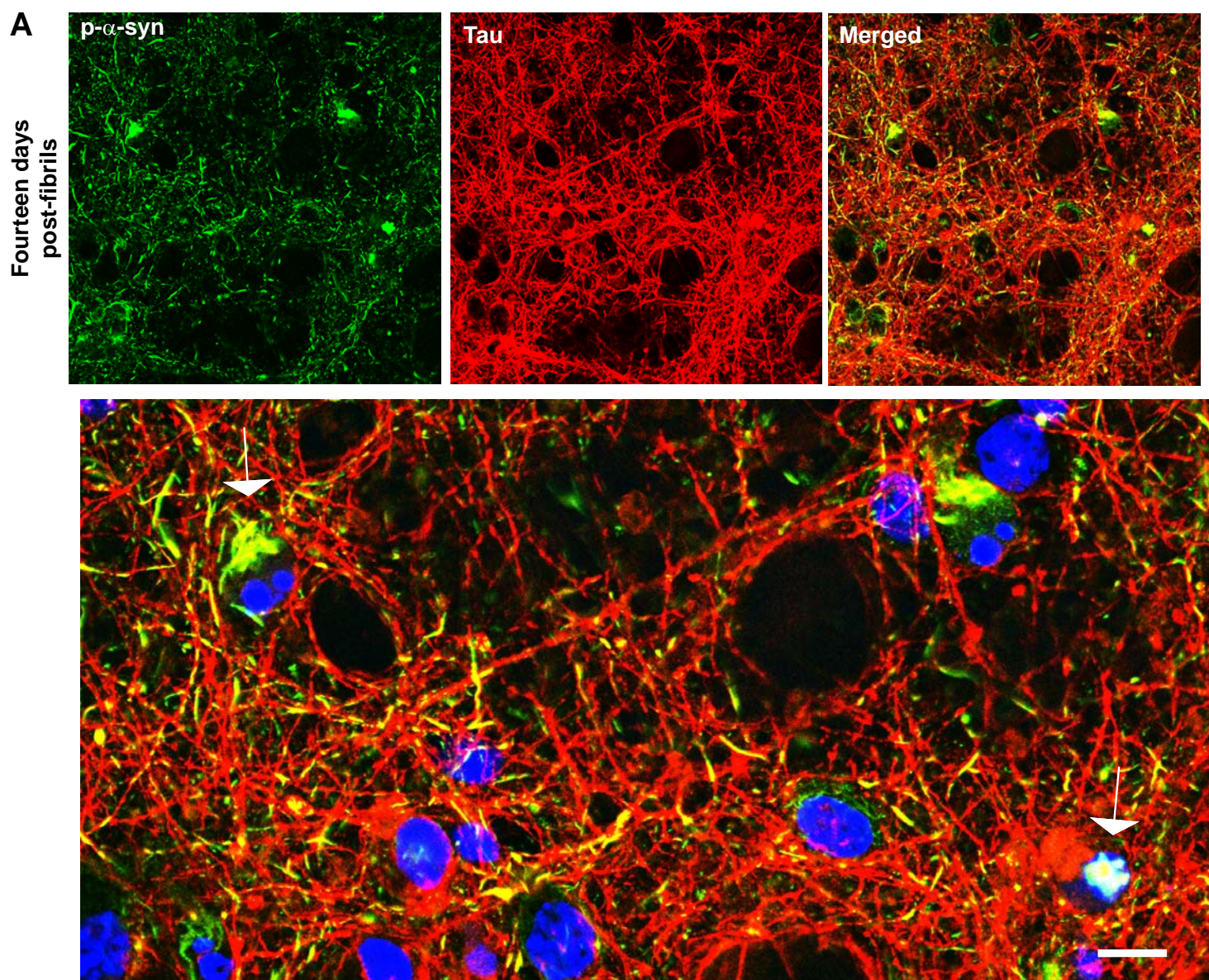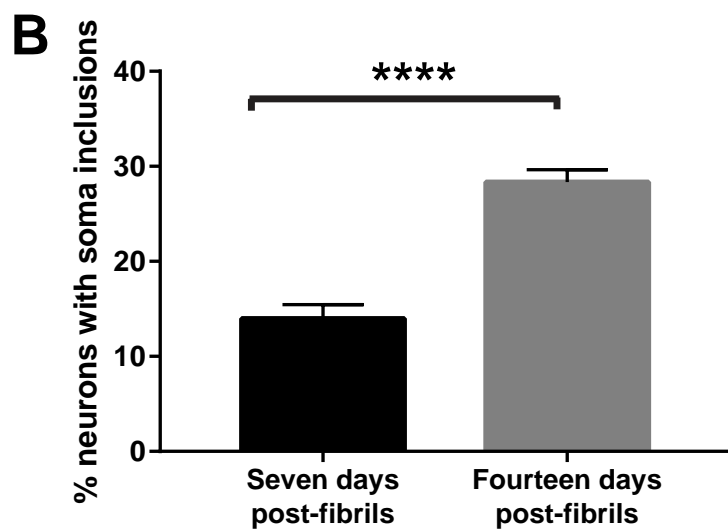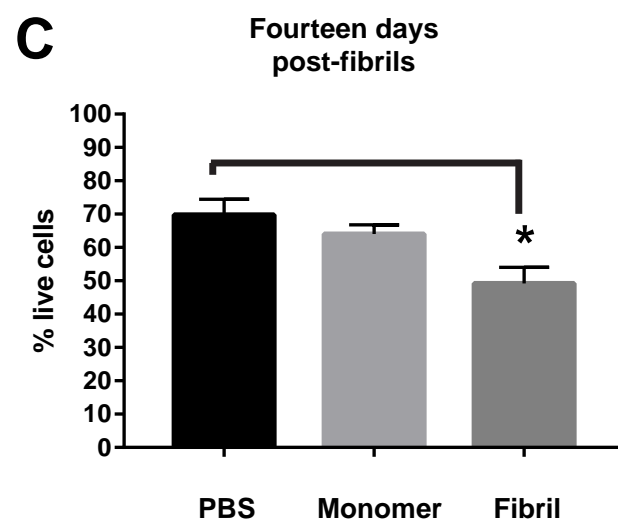

Additional file 2: Figure S2

Supplement: Supplementary file 2 — Figure S2. α-Syn fibrils (2 μg/mL) were added to primary hippocampal neurons on DIV 7 and neurons were fixed 14 days later. A. Immunofluorescence was performed using antibodies to p-α-syn (mouse antibody, green) to label inclusions, tau (red) to label axons, or Hoechst (blue) for nuclei. Images were captured with a confocal microscope at an optical thickness of 0.5 μm. Scale bar = 10 μm. Arrows point to examples of p-α-syn inclusions in the soma. B. The percentage of cells with inclusions near the nucleus was quantified (N = 9). Independent t-test t = 7.1, p < 0.0001. C. Primary neurons were exposed to 2 μg/mL monomer, 2 μg/mL fibrils or PBS at DIV 7. Fourteen days later, calcein AM was used to label live cells and ethidium homodimer-1 was used to label dead cells. Each well was scanned and tiled at 10X. Image J was used to quantify live and dead cells. A total of 40,663 PBS treated cells, 42,348 monomer treated cells, and 45,271 fibril treated cells were counted in two independent experiments. Data is expressed as the average live cells/total number of cells (sum of calcein positive and ethidium homodimer positive). p = 0.864 by ANOVA. (PDF 534 kb) [file 40478_2018_537_MOESM2_ESM.pdf]

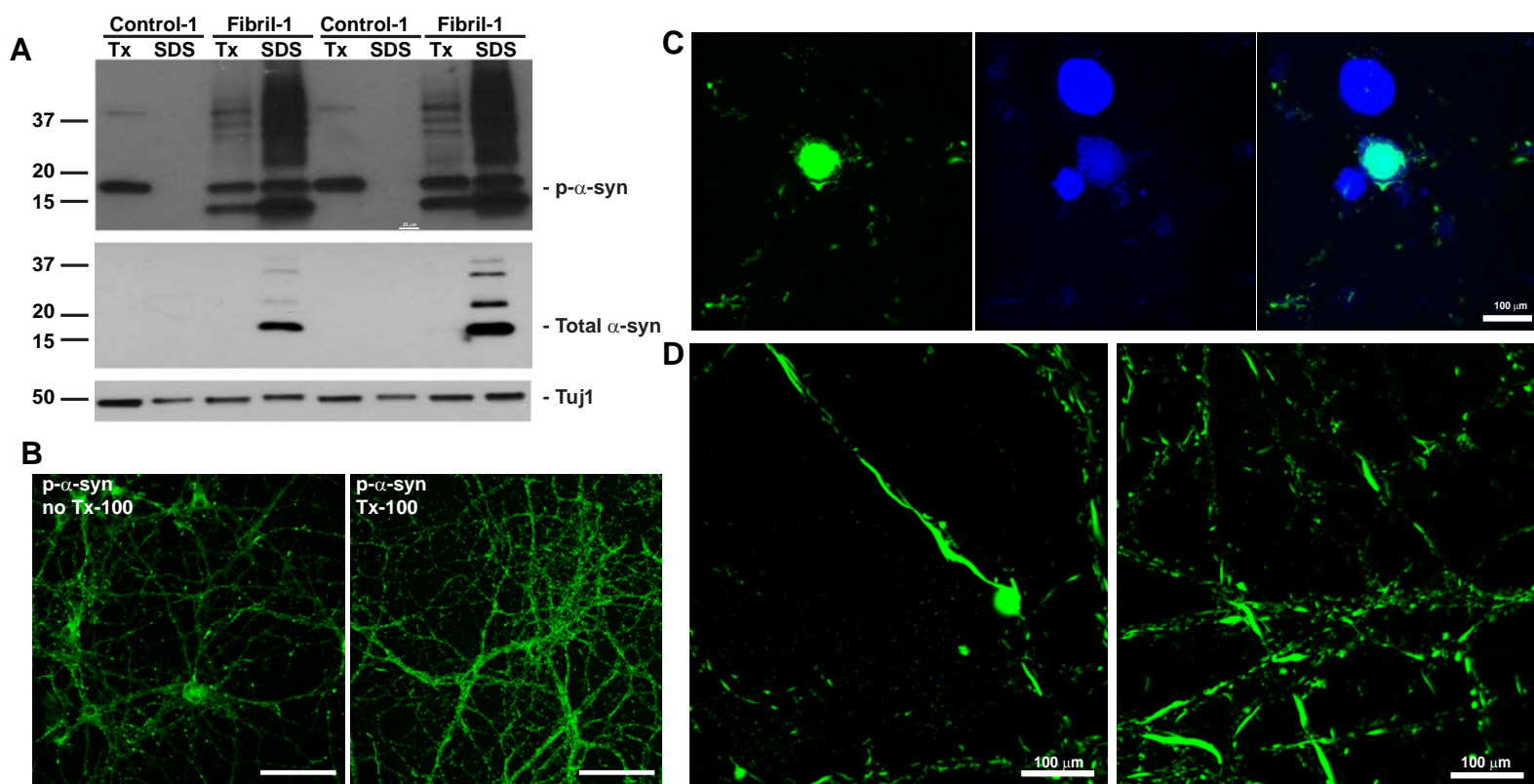

Additional file 3: Figure S3

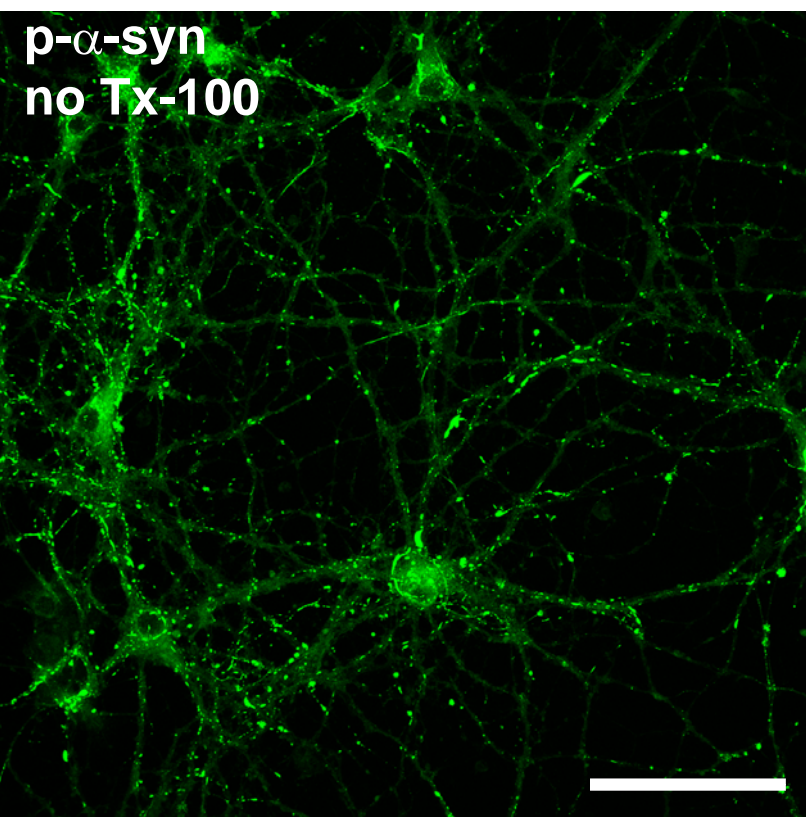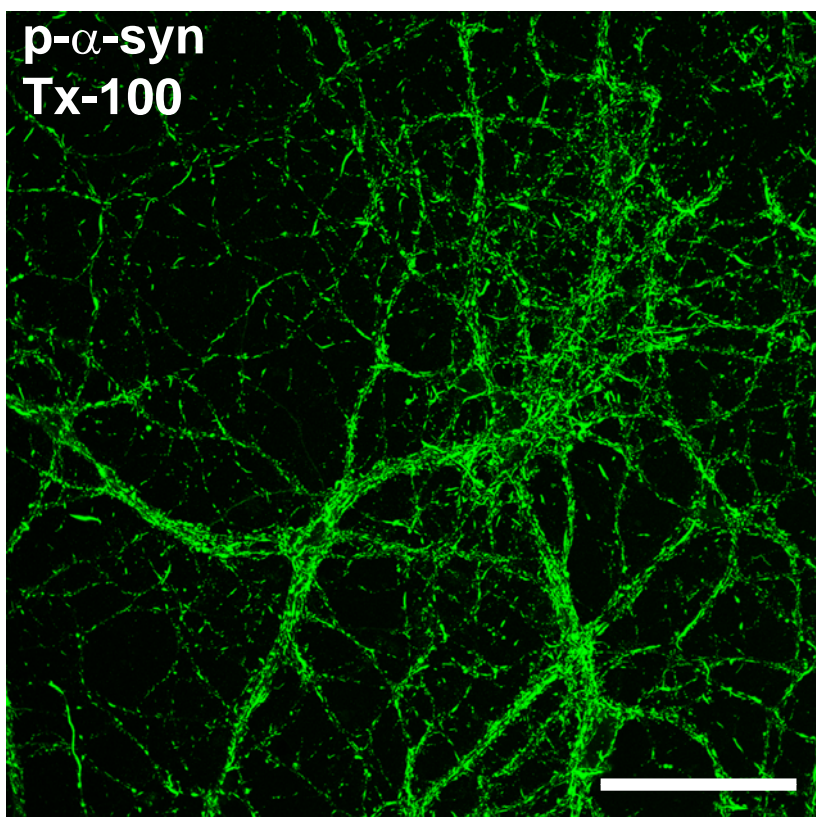

Supplement: Supplementary file 3 — Figure S3. A. Neurons were exposed to fibrils or PBS as a control and were sequentially extracted in 1% Tx-100 followed by 2%SDS. Lysates were subjected to SDS-PAGE on a 4–20% gel and immunoblots were performed with antibodies to p-α-syn, total α-syn or Tuj1 as a loading control. B. Neurons were exposed to fibrils and either fixed with 4% paraformaldehyde (left panel) or 4% paraformaldehyde with 1% Tx-100 (right panel). Immunofluorescence was performed with an antibody to p-α-syn. C. Confocal image of a dense spherical inclusion labeled using an antibody to p-α-syn. Hoechst shows the presence of nuclei, although the nucleus juxtaposed to the inclusion appears fainter compared to the healthier nuclei nearby. D. Examples of aggregates that appear morphologically similar to Lewy neurites. Scale bar = 100 μm. (PDF 919 kb) [file 40478_2018_537_MOESM3_ESM.pdf]

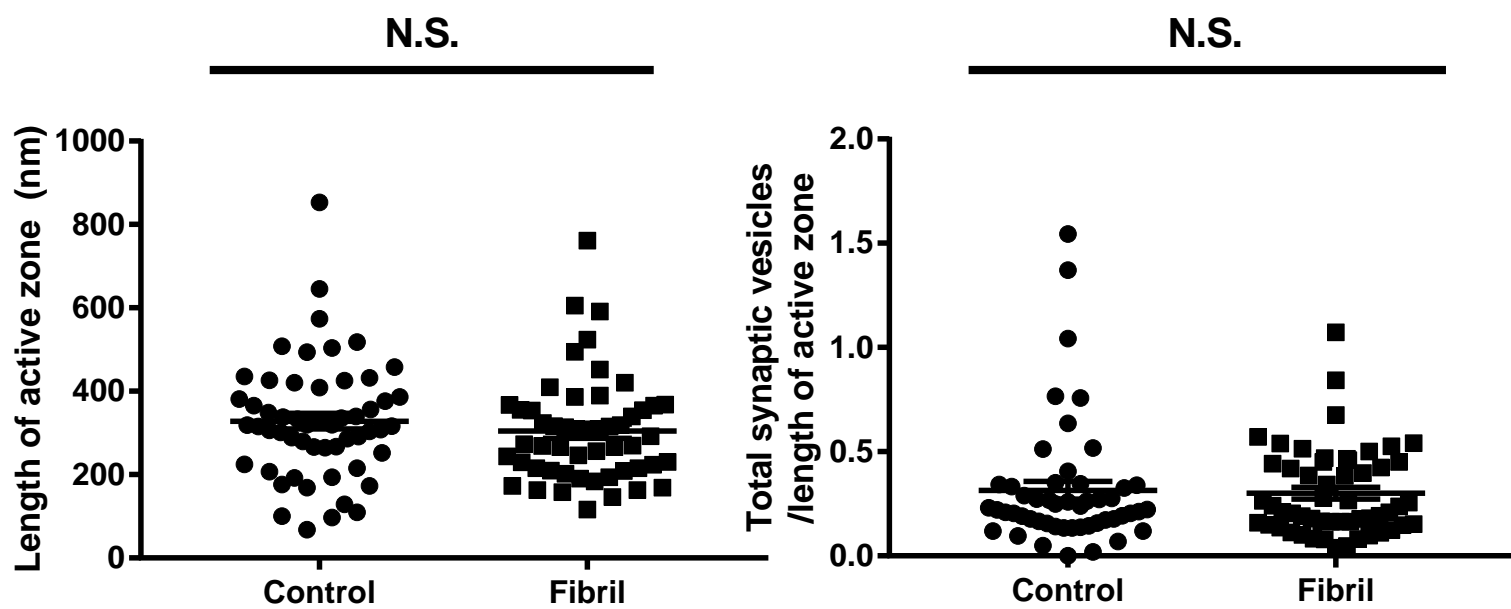

Additional file 4: Figure S4

Supplement: Supplementary file 4 — Figure S4. The length of the active zone and number of synaptic vesicles normalized to active zone length were quantified using transmission electron microscopy images. Only asymmetric synapses were quantified. (PDF 188 kb) [file 40478_2018_537_MOESM4_ESM.pdf]

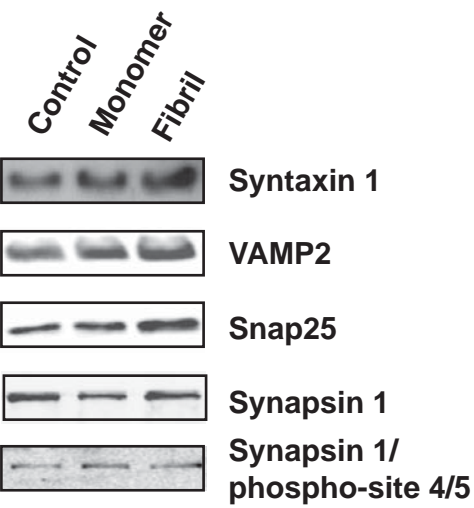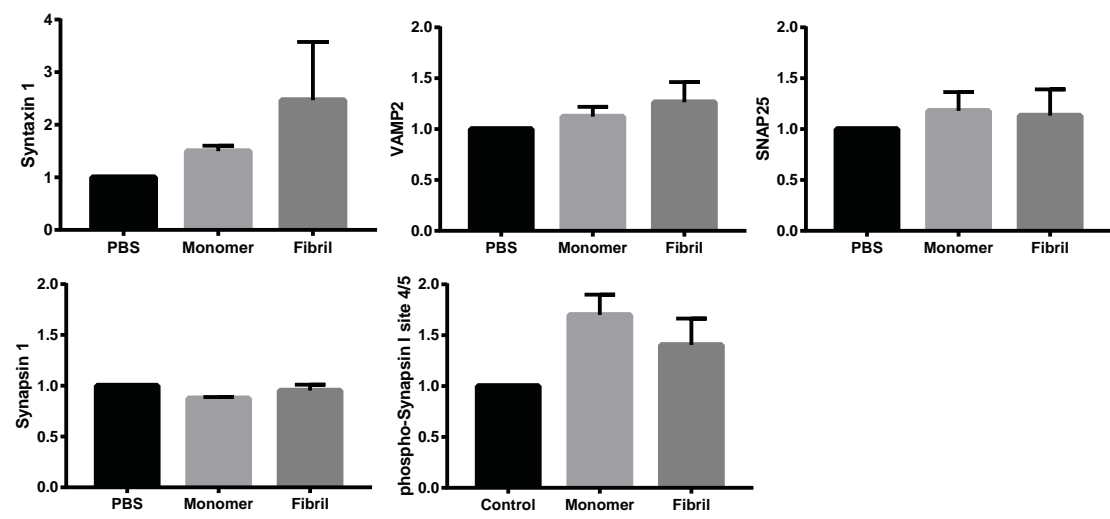

Additional file 5: Figure S5

Supplement: Supplementary file 5 — Figure S5. Primary neurons were either untreated, treated with monomeric α-syn or α-syn fibrils. Seven days later cells were lysed and immunoblots were performed for total levels of syntaxin 1, VAMP2, SNAP25, Synapsin 1, or phospho-Synapsin 1 (site 4/5). The quantitation on the right shows levels of each protein normalized to loading control (vinculin for synapsin 1 and Synapsin 1 for other proteins). The control and fibril exposed neurons represent 6 independent experiments and the monomer exposed neurons represent 3 experiments. There were no significant differences by independent t-test. (PDF 134 kb) [file 40478_2018_537_MOESM5_ESM.pdf]

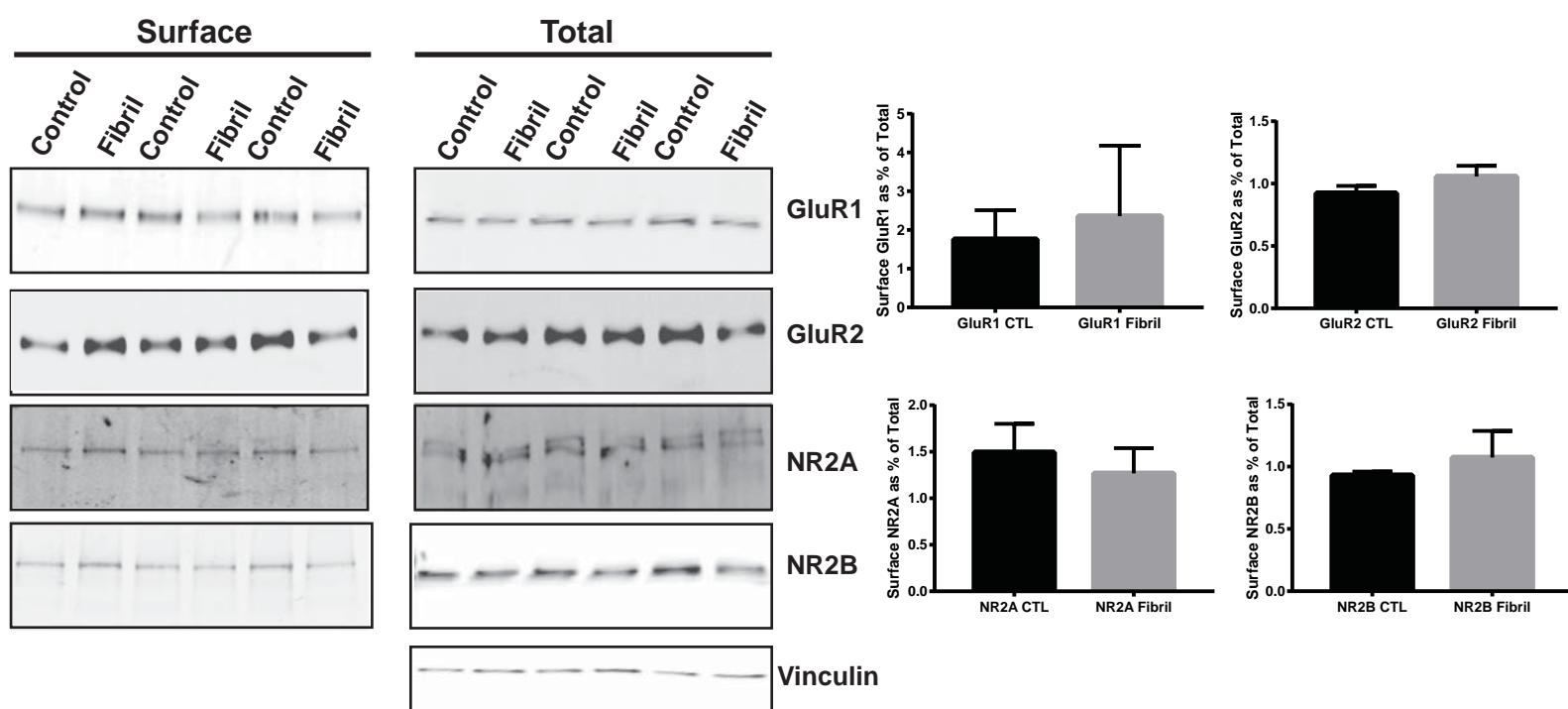

Additional file 6: Figure S6

Supplement: Supplementary file 6 — Figure S6. Primary hippocampal control neurons and neurons with inclusions (7 days post-fibril exposure) were incubated with biotin, lysed, and cell surface proteins were pulled down with neutravidin beads. The left immunoblots show cell surface NR2A, NR2B NMDA receptor subunits and GluR1 and GluR2 receptor subunits. The immunoblots on the right show total levels of each protein. Vinculin was included to demonstrate equal loading. Quantitation on the right from 6 independent experiments show the mean level of surface receptor subunits normalized to total levels of each protein. There were no significant differences by independent t-test. (PDF 129 kb) [file 40478_2018_537_MOESM6_ESM.pdf]
